# Validating Impedance/pH Sensors for Measuring Oesophageal Transit: *A Study Based on Dysphagia and Barium Swallow*

**DOI:** 10.3390/s25113334

**Published:** 2025-05-26

**Authors:** Ismail Miah, Terry Wong, Sebastian Zeki, Jafar Jafari

**Affiliations:** 1 Oesophageal Laboratory, Guy’s & St. Thomas’ NHS Trust, London SE1 9RT, UK; ismail.miah@gstt.nhs.uk (I.M.);; 2 Faculty of Life Science & Medicine, King’s College London, London WC2R 2LS, UK

**Keywords:** oesophagus, transit, impedance, physiology, barium swallow, gastroenterology, general surgery

## Abstract

(1) Background: This study validates multichannel impedance/pH (MII/pH) sensors to measure oesophageal impedance transit (EZT). (2) Methods: EZT involved patients rapidly drinking 200 mL of saline during their MII/pH test. During the EZT study, the oesophageal pH sensor was used to exclude gastric acid reflux occurring and interfering with the oesophageal transit. EZTs were compared between (i) asymptomatic and symptomatic patients with dysphagia and (ii) barium swallow study outcomes for normal oesophageal transit and retention. Statistical *t*-tests, chi-squared tests, receiver operating characteristic curves with Youden’s J Index and regression analysis were conducted. (3) Results: A total of 458 patients (265 females) undertook the transit test during their MII/pH test. Prolonged EZT was found in patients with symptomatic dysphagia (*t*-statistics 4.28–4.43, *p* < 0.001) with the cut-off threshold at 1 min in the distal oesophagus for dysphagia symptoms (sensitivity 0.81, specificity 0.75). EZT was significantly higher in patients with retention on the BS test (*t*-statistics 7.29–8.91, *p* < 0.001), with the distal oesophageal cut-off threshold at 3.7 min being predictive for retention (sensitivity 0.79, specificity 0.93). Increased EZT in the distal oesophagus showed a direct positive correlation to higher dysphagia severity (r = 0.67, *p* < 0.001). (4) Conclusions: MII/pH sensors provide a platform to measure oesophageal transit, which was able to explain dysphagia from poor oesophageal clearance and predict the BS test outcome.

## 1. Introduction

Multichannel impedance/pH (MII/pH) catheters have been commercially available for more than two decades for clinically investigating gastric reflux and objectively diagnosing gastro-oesophageal reflux disease. The MII/pH catheters are a hybrid of multiple impedance sensors integrated into the conventional pH sensor catheter that was traditionally used for reflux monitoring. The impedance sensors distributed along the catheter permit capturing reflux in the mid and proximal oesophagus during the MII/pH test. The MII/pH sensors concordantly capture the acid reflux. The impedance sensors display the reflux from (i) retrograde flow between the impedance sensors [1,2,3] and (ii) detect temporal changes in the impedance from the different alternating current between oesophageal mucosa (2102 Ω–4618 Ω) [3,4,5] and gastric acid (≈260 Ω) [3]. The pH sensor detects the oesophageal luminal ambient pH and the potency of the gastric reflux occurring (acid, weak acid, or non-acid) [1,6].

The series of impedance sensors can also capture the antegrade flow of the oesophageal acid clearance and the bolus clearance from swallows [1,2]. The oesophageal bolus clearance has been referred to by various terms in the literature, including bolus exposure time, bolus contact time, or bolus clearance time [2,6,7,8,9,10]. The ability for impedance sensors to the measure oesophageal bolus clearance raises the potential to measure the oesophageal impedance transit (EZT). This has not been explored in the clinical context of dysphagia symptoms from poor oesophageal clearance nor compared with the standard oesophageal transit testing method (barium swallow [BS] study). The literature has documented the impedance bolus transit from swallows of food bolus to be between 11 to 30 s [3,6,10] or a total of 1.4% impedance bolus exposure in the oesophagus within 24 h [1]. Interestingly, the impedance capture of oesophageal acid clearance is reported to take twice as long (up to 63 s) [11].

The aim of this study is to validate the use of MII/pH sensors for measuring oesophageal transit. Multiple impedance sensors will be used within the oesophageal segments to determine EZT in the proximal, mid and distal oesophagus. The oesophageal pH sensor will be used to exclude gastric reflux interreference occurring the during the EZT test. The primary objective is to perform EZT testing during the MII/pH test, and the validation of MII/pH is accomplished by differentiating EZT in patients asymptomatic and symptomatic of dysphagia and correlating EZT to the standard BS test. The secondary objective is to determine EZT thresholds for poor oesophageal clearance leading to dysphagia symptoms and predicting oesophageal contrast retention (OCR) on the BS test.

## 2. Materials and Methods

### 2.1. Participant Selection and Ethical Approval

The participants in this study were patients with clinical referrals for upper GI endoscopy, BS study, high-resolution manometry (HRM) and MII/pH tests, which was part of their standard diagnostic testing for dysphagia and/or reflux symptoms. The EZT test was performed during the MII/pH test and the MII/pH test was performed prospectively to the upper GI endoscopy and the BS studies (see Table 1 for the study inclusion and exclusion criteria). The gastroscopy and x-ray imaging of the oesophagus were used to exclude patients with oesophagitis, oesophageal mass/cancer and diverticulum. The study exclusion criteria mirror the pre-requisites that would be expected in healthy volunteers.

**Figure 1:**
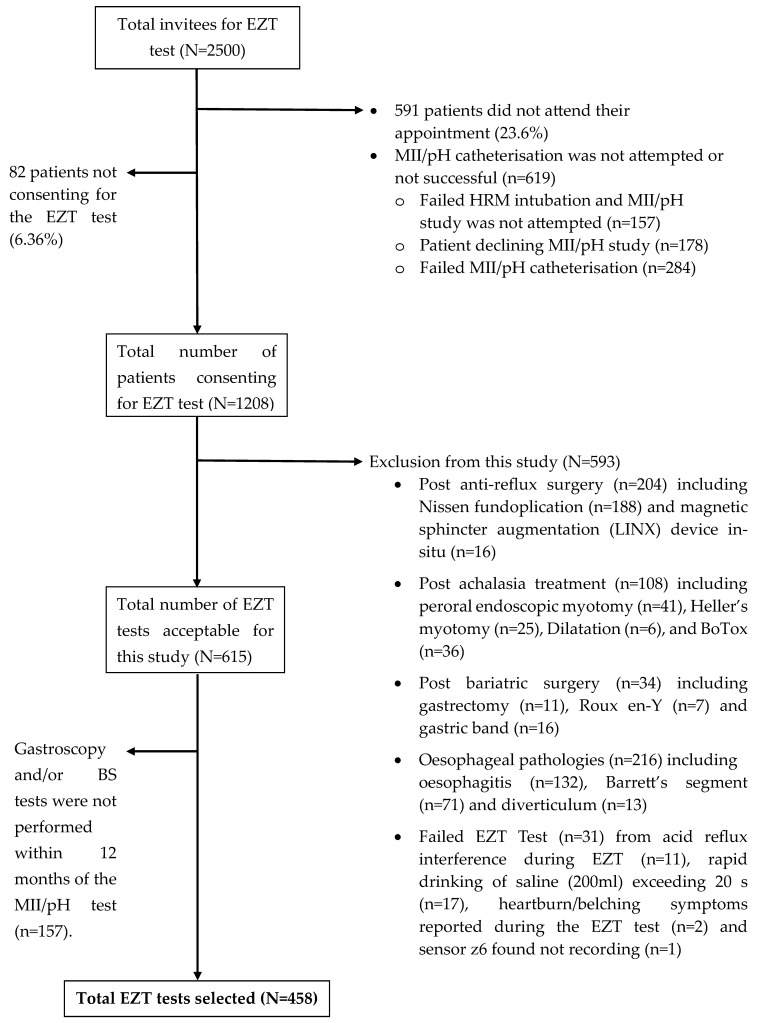
Flow chart for selecting patients for this study.

The patient referrals for the MII/pH study were vetted with respect to the inclusion criteria in Table 1. This was then used to invite patients for the EZT study. The patient information sheets of the EZT study were sent out to 2500 patients along with their appointment letter for the MII/pH test. A total of 1909 of the 2500 patients (76.4%) attended their appointment (see Figure 1). During their appointment, a thorough clinical history was taken, and obtaining consent for the MII/pH test and the EZT test was appropriately conducted. During the clinical tests, 619 patients (32.4%) did not tolerate the HRM test or the MII/pH catheterisation. Of the 1290 patients successfully undertaking the MII/pH study (51.6%), 82 patients did not consent to the EZT study (6.36%). Of the 1208 patients who undertook the EZT test, 593 EZT studies were excluded because patients had histories of anti-reflux surgery (34.4%), oesophageal treatment for achalasia (18.2%), bariatric surgery (5.73%) and oesophageal pathologies (36.4%) outlined in the exclusion criteria (see Table 1). Thirty-one patients failed the EZT tests (5.23%). Finally, 615 EZT tests could be used for this study, but 157 patients (25.5%) did not have the gastroscopy and/or BS studies within the 12 months of the MII/pH test. Therefore, the EZT tests of only 458 patients were included in this study.

The participants of this study were patients with clinical referrals for the relevant upper GI investigations and, therefore, this research project fulfilled the declaration of Helsinki. The research protocol was approved by the Institutional Gastroenterology and GI Surgery Review Board. This study is also under the umbrella of a wider clinical research, which is approved by the Research Ethics Committee (reference 18/NW/0120, IRAS ID 333800). The EZT tests were conducted between January 2020 and December 2023, and all the patients were given the HODQ form as part of their HRM testing. The HODQ form was used to evaluate the severity of dysphagia in patients [12].

### 2.2. MII/pH Catheterisation and Impedance/pH Sensor Positioning

**Figure 2:**
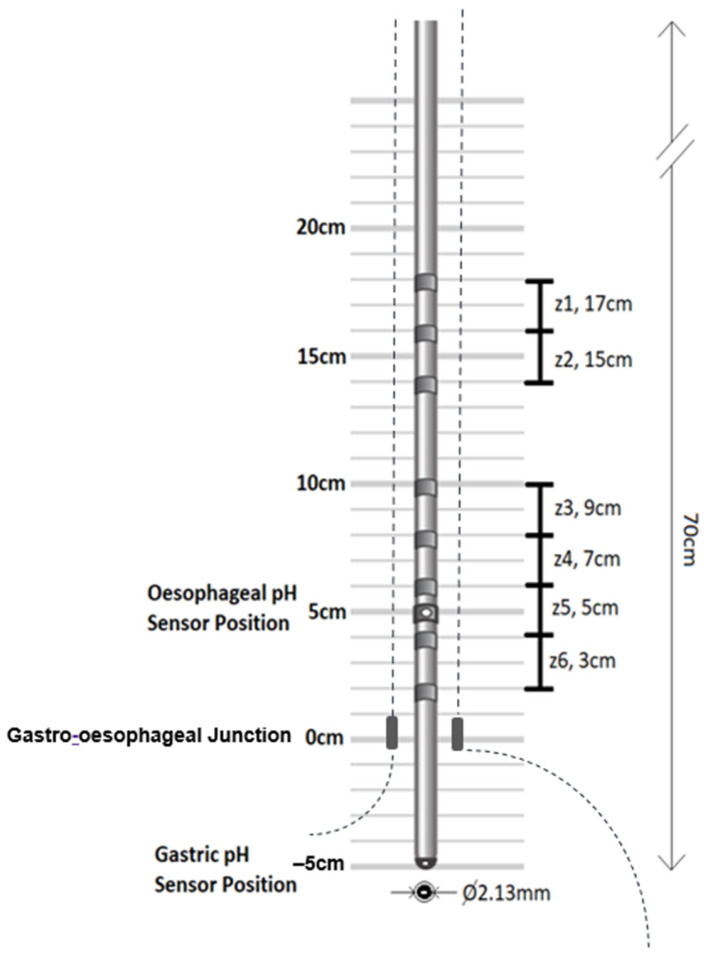
Schematic diagram of the MII/pH catheterisation and arrangement of the impedance and pH sensors within the gastro-oesophageal lumen (catheter reference ZAI-BG-44, manufactured by Diversatek Healthcare, Highlands Ranch, CO, USA). The impedance electrodes are positioned at 3 cm, 5 cm, 7 cm, 9 cm, 15 cm and 17 cm above the LOS, and the pH sensors were positioned 5 cm above and below the manometric GOJ.

Local anaesthesia (xylocaine spray) was administered into one side of the nose (3–5 sprays, each spray containing lidocaine 30 mg) (Aspen Pharma Trading Limited, Dublin 24, Ireland). The distal portion of the MII/pH catheter was smeared with lubrication gel (approximately 5 cm from the catheter tip), and the catheter was inserted into the anaesthetised nostril to assess patient toleration and resistance to passage through the nasal cavity. If the nasal intubation was successful, patients were asked to drink water, and the catheter was slowly advanced into the cricopharyngeus and then into the oesophagus. The intubation was continued into the stomach by additional swallowing manoeuvres until the intubation depth was approximately 50 cm and the distal pH sensor was capturing acidic pH readings of the stomach (pH < 4). The MII/pH catheter was then re-adjusted to position the oesophageal pH sensor to 5 cm above the manometric GOJ, which registered neutral pH of the oesophagus [13]. The distal pH sensor was automatically positioned 5 cm below the manometric LOS, which was confirmed by measuring the gastric acidic pH. The catheter extension was looped over the patient’s ear on one side of the face, which was on the side of the nasal catheterisation. The catheter was taped down on the cheek and neck. The positioning of pH sensors takes priority for the MII/pH study to capture the distal oesophageal acid reflux. In this positioning of the oesophageal pH sensor, the impedance sensors were automatically arranged and positioned to capture impedance at 3 cm (z6), 5 cm (z5), 7 cm (z4), 9 cm (z3), 15 cm (z2) and 17 cm (z1) above the manometric GOJ (see Figure 2). The oesophageal pH sensor was used during the EZT test to assess for gastric reflux occurring and interfering with the oesophageal transit.

### 2.3. The EZT Test Protocol

The MII/pH catheter was intubated and positioned as described in Section 2.2. The EZT test was conducted within the first 10 min of the MII/pH test and was marked as the first meal event on the recording. The patients were seated in the upright position and were requested to inhale and rapidly drink 200 mL of saline within 20 s (exhalation was permitted between swallows if required). The saline volume and drinking protocol were adopted from the study by Cho and colleagues [9] who conducted an oesophageal transit study during the high-resolution manometry with impedance (HRMZ) test. In addition, our patients also drank a 200 mL volume of the barium sulphite contrast for their BS test [8], which is consistent with the saline volume used for the EZT test.

**Figure 3:**
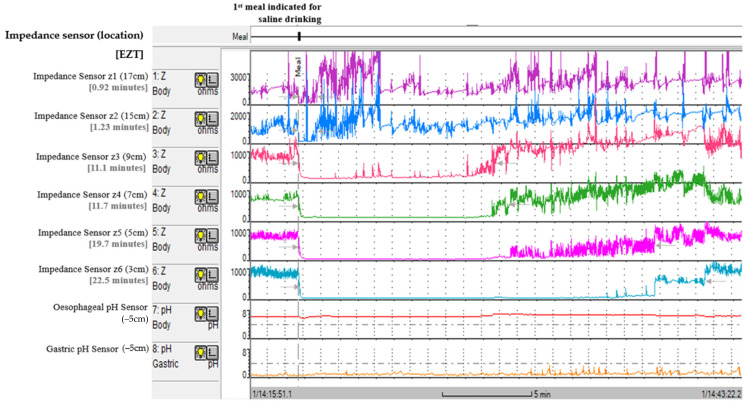
EZT recording in a patient with dysphagia symptoms (HODQ score 26) and OCR was found on the BS test. The saline transit was identified in all the impedance sensors (z1, z2, z3, z4, z5 and z6) from the 50% impedance fall to the 50% impedance rise (see grey arrow marking).

The EZT test on the MII/pH recording presented an antegrade flow pattern from the proximal oesophagus to the distal oesophagus (see Figure 3). The impedance changes were identified by the difference in the alternating current between oesophageal mucosa and saline. When saline cleared from each of the oesophageal segments, the mucosal impedance was observed again. The saline transit duration at each impedance sensor was calculated from the 50% impedance drop from the baseline mucosal impedance to the 50% impedance recovery to the baseline mucosal impedance [3,7] (this is marked by the grey arrows in Figure 3). The saline transit was computed from the impedance sensors z2, z3 and z6, which correspond to the proximal, mid and distal oesophageal segments. The EZT study in Figure 3 corresponds to 1.23 min, 11.1 min and 22.5 min. During this EZT test, the oesophageal pH was stable at neutral, which excludes acid reflux occurring and interfering with the transit test.

### 2.4. Statistical Analysis

The EZT of the proximal, mid and distal oesophageal segments were computed from impedance sensors z2, z3 and z6 (see Section 2.3). The data of the EZT are presented in the median and interquartile range (IQR). The EZT data were compared as follows:

(i) Assessing EZT in patients with respect to normal and elevated HODQ scores for dysphagia and the BS test outcomes for normal contrast transit (NCT) and OCR. Although HODQ scores are defined at point of significant dysphagia (scores > 6.4) [12], a subtest will be undertaken to thoroughly assess the EZT technique from asymptomatic patients (HODQ scores of 0) and patients with minor dysphagia symptoms (HODQ scores from >0 to 6.4).
(ii) Computing EZT thresholds that lead to poor oesophageal clearance and dysphagia symptoms and thresholds to predict OCR on the BS test.
(iii) Comparing the EZT data from the proximal, mid and distal oesophagus to determine the best region for conducting the EZT test.

EZT data comparison was performed using the statistical *t*-test and chi-squared tests (where appropriate). Any *p*-value less than 0.05 was considered statistically significant. Receiver operating characteristic (ROC) curves were plotted, and cut-off thresholds for EZT were computed from the maximum Youden’s index (Youden’s indices greater than 0.50 were considered satisfactory outcomes for models to discriminate between dysphagia severity and predicting BS outcomes for NCT and OCR). The EZT cut-off thresholds at each oesophageal segment were assessed to determine sensitivities and specificities to explain the dysphagia and predict the BS test outcome. Predictive positive value (PPV) and negative predictive value (NPV) probabilities were computed to identify the patients with true dysphagia and true OCR in the BS study at the cut-off thresholds. The likelihood ratio (LR+) and odds ratio (OR) were also calculated to assess the EZT cut-off thresholds to detect significant dysphagia and predict the BS study. C-statistics were computed from the area under the curve to assess the model to discriminate between dysphagia severity, BS outcomes and the oesophageal segments where saline transit was computed.

We performed complementary statistical tests to support the validation of the findings. This included regression analysis of the median EZT of the distal oesophagus and the dysphagia severity. The linear regression best-fit function of the EZT to HODQ scores was used to confirm EZT points of (i) poor oesophageal clearance leading to dysphagia and (ii) the point of predicting OCR on BS study outcome. Subtests of the linear regression between EZT and HODQ scores were performed at normal and elevated ranges of the HODQ scores. This was to assess EZT data falling within normal regions, the point of dysphagia occurring and the point of OCR in the BS study. The BS study is a qualitative assessment of NCT or OCR, and logistic regression statistics were employed along with odds assessment for the BS test outcome to determine the validity of the EZT cut-off threshold for OCR.

## 3. Results

### 3.1. Patient Demography

A total of 1208 patients participated in this study from invitations to 2500 patients (48.3%). A total of 458 of the 1208 patient participants (37.9%) fulfilled the inclusion and exclusion criteria (female/male 265:193, ages 19 to 88 years old). A total of 419 patients (91.5%) successfully completed the HODQ form (39 forms were incomplete with missing answers). A total of 256 of the 419 patients (61.1%) had elevated HODQ scores for significant dysphagia, and 147 of the 458 patients (32.1%) demonstrated OCR in the BS study. A total of 107 patients of the 147 (72.8%) with OCR on the BS test also had elevated HODQ scores for significant dysphagia, while 144 of the 311 patients (46.3%) with NCT on the BS study had normal HODQ scores (χ^2^ = 15.14, *p* < 0.001). Concordance for a normal or elevated HODQ score to NCT or OCR, respectively, on the BS test was found in 251 patients (59.9%). The HODQ scores found for patients with NCT on the BS (median 7, SQR [2.0–14.0]) and OCR on the BS (median 19, SQR [12.0–32.5]) were statistically different (*t*-statistic = 9.27, *p =* 0.004).

### 3.2. The Validation of EZT Test

The EZT in patients with normal and elevated HODQ scores and the BS test outcomes (NCT or OCR) are outlined in Table 2. The EZT is significantly longer in patients with elevated HODQ scores, which was observed in the proximal oesophagus (0.47 min vs. 0.23 min, *t*-statistic = 4.28, *p* < 0.001), mid oesophagus (0.85 min vs. 0.31 min, *t*-statistic = 4.05, *p* < 0.001) and distal oesophagus (1.90 min vs. 0.57 min, *t*-statistic = 4.43, *p* < 0.001). The EZT was also statistically longer in the patients with OCR on the BS test, which was observed in the proximal oesophagus (5.75 min vs. 0.26 min, *t*-statistic = 7.29, *p* < 0.001), mid oesophagus (15.4 min vs. 0.36 min, *t*-statistic = 7.43, *p* < 0.001) and in the distal oesophagus (22.8 min vs. 0.72 min, *t*-statistic = 8.91, *p* < 0.001). The *t*-statistic values are highest for the distal oesophagus in comparison to the mid and proximal oesophagus when assessing for dysphagia from asymptomatic patients or OCR from NCT on BS test outcome. This suggests the greatest difference in EZT is in the distal oesophagus, which is therefore statistically superior.

When comparing EZT between patients with OCR in the BS test and patients with elevated HODQ scores, the findings show a statistically higher transit time for patients with OCR in the proximal oesophagus (5.75 min vs. 0.47 min, *t*-statistic = 3.55, *p* < 0.001), mid oesophagus (15.4 min vs. 0.85 min, *t*-statistic = 3.65, *p* < 0.001) and in the distal oesophagus (22.8 min vs. 1.90 min, *t*-statistic = 4.30, *p* < 0.001). This suggests that patients with OCR have a much higher severity of dysphagia than of a HODQ score of 6.4. It is also noticeable that patients with elevated HODQ scores had significantly higher EZT compared to a cohort of patients with NCT in BS studies. This was observed in the proximal oesophagus (0.47 min vs. 0.26 min, *t*-statistic = 5.85, *p* < 0.001), mid oesophagus (0.85 min vs. 0.36 min, *t*-statistic = 6.01, *p* < 0.001) and in the distal oesophagus (1.90 min vs. 0.72 min, *t*-statistic = 6.76, *p* < 0.001). Finally, the EZT between patients with NCT on the BS test was found to be similar to the EZT found in patients with normal HODQ scores. This is consistently seen in the proximal oesophagus (0.26 min vs. 0.23 min, *t*-statistic = 1.48, *p =* 0.140), mid oesophagus (0.36 min vs. 0.31 min, *t*-statistic = 1.31, *p =* 0.193) and in the distal oesophagus (0.72 min vs. 0.57 min, *t*-statistic = 1.60, *p =* 0.110).

A total of 163 patients (38.9%) had normal HODQ scores (scores ≤ 6.4). Of which, 67 patients (41.1%) were completely asymptomatic of dysphagia (HODQ score 0), while 96 patients (58.9%) had minor dysphagia symptoms (HODQ scores greater than 0 and up to 6.4). The asymptomatic patients demonstrated EZT in the proximal, mid and distal oesophagus, respectively, to 0.20 min (SQR 0.12–0.35), 0.26 min (SQR 0.14–0.49) and 0.40 min (SQR 0.21–0.80), which are statistically lower compared to the EZT found in patients with minor dysphagia symptoms (proximal oesophagus: 0.20 min vs. 0.26 min, *t*-statistic = 2.07, *p =* 0.040; mid oesophagus: 0.26 min vs. 0.31 min, *t*-statistic = 2.22, *p =* 0.028; and distal oesophagus: 0.40 min vs. 0.60 min, *t*-statistic = 2.41, *p =* 0.017). The EZT in patients asymptomatic of dysphagia, patients with minor dysphagia and patients with significant dysphagia with respect to the BS test outcomes can be found in Table 3. Notably, only the asymptomatic patients had no statistical difference in EZT despite different BS test outcomes, and patients with minor and significant dysphagia symptoms had statistical differences in the EZT with respect to the BS test outcome. In detail, in the asymptomatic patients (n = 67), five patients (7.46%) revealed OCR on the BS test, but their EZT was not statistically different from the patients with NCT (*t*-statistic = 0.214–0.349, *p* > 0.05). In the cohorts of patients with minor dysphagia (n = 96), eight patients (8.33%) showed OCR on the BS test, which is statistically higher in EZT compared to patients with NCT in the BS studies. This was observed in the proximal oesophagus (2.00 min vs. 0.23 min, *t*-statistics = 3.62, *p* < 0.001), mid oesophagus (3.85 min vs. 0.30 min, *t*-statistics = 2.16, *p =* 0.033) and in the distal oesophagus (13.1 min vs. 0.52 min, statistics = 3.19, *p =* 0.002). In the cohort of patients with significant dysphagia (n = 256), larger portions of patients had OCR in the BS test (134 patients, 52.3%). The patients with OCR on the BS test also showed statistically higher EZT in the proximal oesophagus (6.70 min vs. 0.28 min, *t*-statistic = 6.63, *p* < 0.001), mid oesophagus (18.8 min vs. 0.48 min, *t*-statistic = 7.00, *p* < 0.001) and in the distal oesophagus (26.3 min vs. 1 min, *t*-statistic = 8.29, *p* < 0.001).

### 3.3. EZT Cut-Off Thresholds for Dysphagia

The maximum Youden’s index for discriminating between normal and elevated HODQ scores for dysphagia was positive only in the distal oesophagus (0.56). Youden’s indices showed poor classification for the proximal oesophagus (0.33) and mid oesophagus (0.36) (see Table 4 for details). The EZT cut-off threshold at the distal oesophagus at the maximum J index (1 min) captured 81% of patients with elevated HODQ scores, whilst true values were 84%. When EZT was less than 1 min in the distal oesophagus, this captured 75% of patients with normal HODQ scores, whilst 72% truly had normal HODQ scores. The EZT cut-off threshold found for the proximal oesophagus at the maximum Youden’s index (0.47 min) was only able to identify 50% of the patients with elevated HODQ scores, and the EZT cut-off threshold for the mid-oesophagus at the maximum Youden’s index (0.66 min) detected 57% of the patients with significant dysphagia.

At the cut-off thresholds of the oesophageal segments, the PPV probability for detecting elevated HODQ scores was marginally different, but the highest was found for the distal oesophagus (84%). The NPV was also the highest for the EZT of the distal oesophagus (72%) compared to the proximal oesophagus (53%) and mid oesophagus (55%). The LR+ and the OR for the EZT cut-off threshold for capturing or excluding significant dysphagia (HODQ scores > 6.4) were also the highest for the distal oesophagus (LR+ 3.32, OR 13.6). The ROC curve model’s *c*-statistics were also marginally higher for the EZT test for the distal oesophagus.

The Youden’s indices to distinguish between patients asymptomatic of dysphagia and minor dysphagia were low in the proximal oesophagus (0.19), mid oesophagus (0.16) and distal oesophagus (0.20) (see Table 5). The low specificities (30–34%) produce false positives of minor dysphagia in many patients. This may be interpreted as patients with HODQ scores of 0 and up to 6.4 have similar or overlapping degrees of dysphagia, which can be non-existent to a minor form. The PPV and NPV were similar, correctly predicting near to half of the time having minor or no dysphagia.

### 3.4. EZT Test Correlation to the BS Test Outcome

The patient demography in this study with NCT on the BS test (n = 311) demonstrated a median HODQ score of 7 (SQI 2 to 14), with 167 patients (53.7%) having HODQ scores greater than 6.4. This suggests approximately half of the patients with NCT in the BS study have significant dysphagia symptoms and would be diagnosed with functional dysphagia. The EZT of the distal oesophagus in 107 of the 167 patients (64.1%) exceeded 1 min, which may explain the dysphagia symptom from poor oesophageal clearance. The patients with OCR in the BS study have significantly higher HODQ scores (median 19, SQR [10–32]) (*t*-statistic = 9.27, *p* < 0.001), and 127 of the 147 patients with OCR had HODQ scores > 6.4 (86.4%). In the 20 patients with OCR on the BS test and scoring less than 6.4 on HODQ, 15 patients (75%) revealed the EZT of the distal oesophagus to be less than 1 min.

The EZT in patients with OCR on the BS test was significantly higher than in patients with NCT in BS studies (see Section 3.2). This revealed similar *t*-statistic values for the oesophageal segments (proximal 7.29, mid 7.43 and distal 8.91). The Youden’s indices were positive for discriminating between NCT and OCR on EZT from the three oesophageal segments. This was found to be 0.58, 0.66 and 0.72 in the proximal, mid and distal oesophagus, respectively (see Table 6 for details). Thus, a strong correlation could be observed between the EZT finding and the BS test outcome.

At the maximum Youden’s indices, EZT cut-off thresholds for proximal, mid and distal oesophagus were, respectively, 0.59 min, 1.9 min and 3.7 min for predicting BS test outcomes. Based on the proximal oesophagus, the EZT cut-off threshold captured OCR in 73% of patients, whilst there were 68% with true positives for OCR on the BS test. The detection of NCT on the BS test when EZT is less than 0.59 min in the proximal oesophagus was observed in 85% of patients, whilst 88% were true negatives for NCT. The overall finding of the LR+ (4.89) and OR (15.5) were positive for finding BS test with OCR when the EZT for proximal oesophagus captures greater than 0.59 min.

In the mid oesophagus, the EZT cut-off threshold (1.9 min) detected OCR on the BS test in 74% of the patients, whilst true positives for OCR in the BS test were 80%. EZT findings less than 1.9 min in the mid oesophagus detected 92% with NCT in the BS study, whilst 89% had true negatives at this threshold. The LR+ (9.18) and OR (33.0) were positive for finding OCR in the BS study when the mid oesophagus captures an EZT greater than 1.90 min. Finally, in the distal oesophagus, the EZT cut-off threshold (3.7 min) detected 79% of the patients with OCR on the BS test for true positives in 83% at this threshold. An EZT less than 3.7 min in the distal oesophagus captured 93% of cases with NCT on the BS test for true negatives in 91%. The highest LR+ (10.6) and OR (46.2) probabilities were found for EZT of the distal oesophagus. The *c*-statistics were also marginally higher for the distal oesophagus.

### 3.5. Complementary Statistical Testing

**Figure 4:**
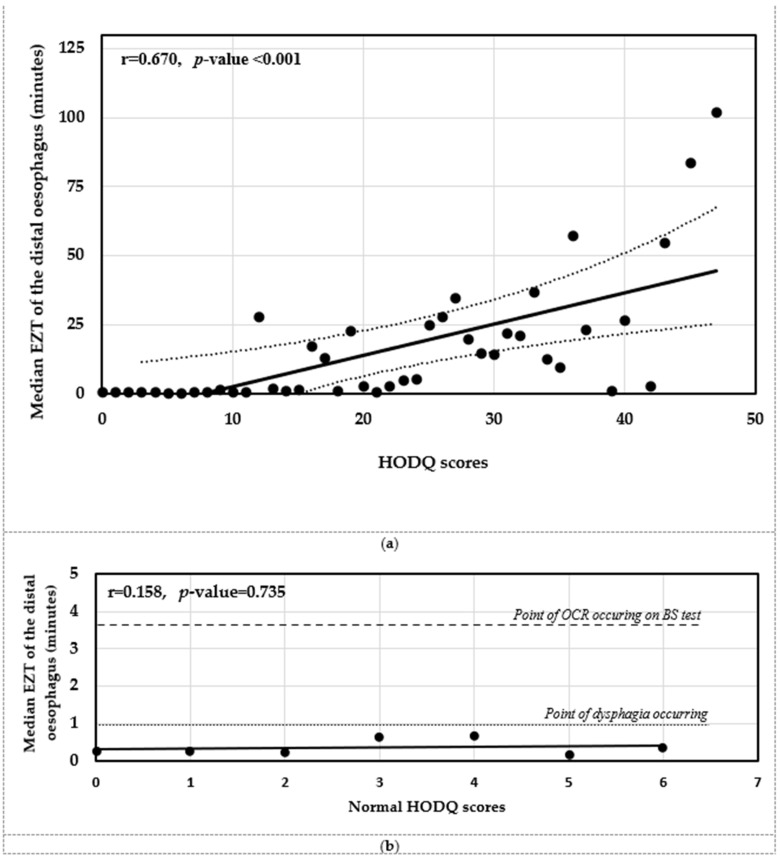

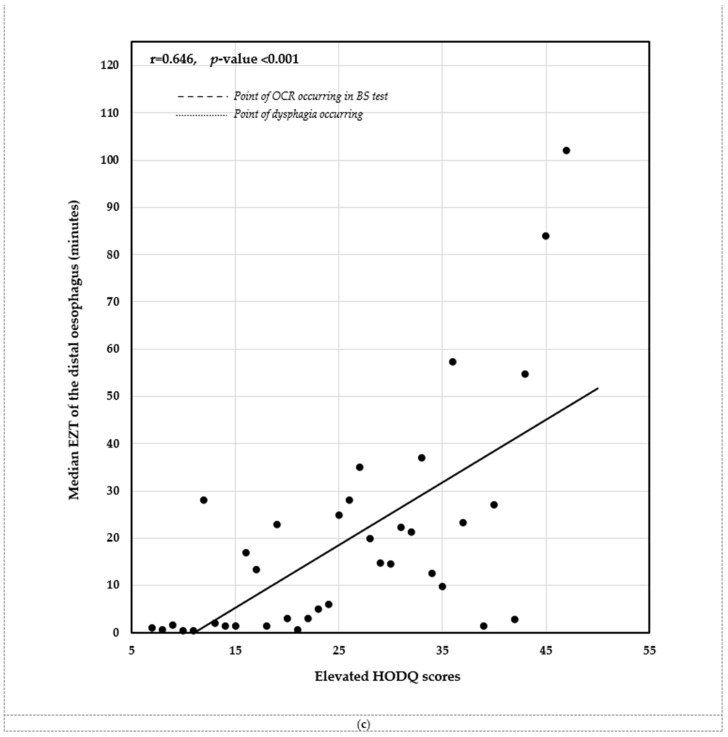
(**a**) Correlation between EZT (median) of the distal oesophagus for dysphagia severity (HODQ scores from 0 to 50) (dashed indicating 5–95% condifence interval of data). (**b**) EZT (median) for normal HODQ score range (from 0 to 6.4) with thresholds at points of dysphagia occurring and OCR occurring on the BS test. (**c**) EZT (median) for positive HODQ scores for dysphagia patients (scores from 6.4 to 50) with thresholds at points of dysphagia occurring and OCR occurring on the BS test.

The Pearson correlation showed direct positive trends for increasing EZT of the distal oesophagus with higher dysphagia severity (r = 0.670, *p* < 0.001) (see Figure 4a). The best-fit linear regression trend was computed as EZT = 1.13 × [HODQ score] − 8.49. The overall gradient suggests EZT (per minute) is approximately proportional one-to-one to one unit of dysphagia severity. Implementing the cut-off threshold, 1 min, for poor oesophageal clearance correlates to an elevated HODQ score of 8.4. The cut-off threshold for finding OCR on the BS test at 3.7 min correlates to a much higher HODQ score of 10.8. This finding also supports the primary finding for the poor oesophageal clearance from 1 min causing significant dysphagia and 3.7 min causing higher severity of dysphagia from the retention (i.e., stenosis). Despite a best-fit trend finding a 1:1 ratio of EZT per minute to dysphagia severity observed, the subtest demonstrated interesting findings. The EZT of the distal oesophagus was similar on the normal HODQ scores (0 to 6.4) (r = 0.158, *p* = 0.735) (see Figure 4b). This is consistent with the findings in Section 3.3 when comparing EZT between patients with HODQ scores of 0 (asymptomatic patients) and from scores > 0 to 6.4 (patients with minor dysphagia). More importantly, the 1:1 ratio of the EZT per minute to dysphagia severity trend was observable for the elevated HODQ scores (i.e., from 6.4 to 50) (r = 0.646, *p* < 0.001) (see Figure 4c). The data revealed only five elevated HODQ scorers had normal EZT (13.5%), 32 elevated HODQ scorers had EZT at the point of dysphagia occurring from poor clearance (86.5%) and 22 elevated HODQ scorers had EZT at the point OCR occurring on the BS test (59.5%). Overall, the EZT at the proposed threshold demonstrated good correlation to dysphagia symptoms.

**Figure 5:**
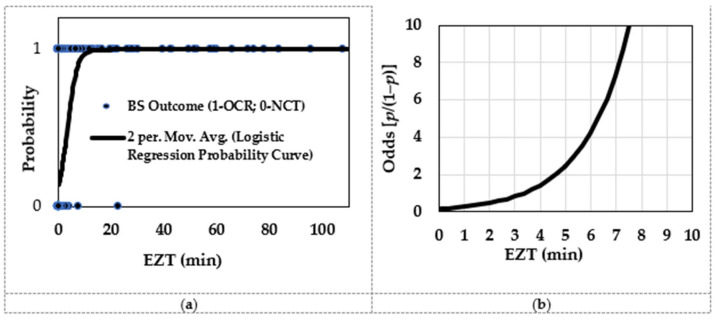
Logistic regression analysis for (**a**) probability curve of BS outcome and EZT of the distal oesophagus and (**b**) the odds of BS outcome for OCR/NCT and EZT of the distal oesophagus.

The EZT threshold of the distal oesophagus to predict the BS test for OCR or NCT revealed greater statistical affinity to the true positives and negatives compared to the EZT thresholds found for the mid oesophagus and proximal oesophagus. This of course generated the highest LR (10.6) and the OR (46.2) for the EZT cut-off threshold of the distal oesophagus, which we believed would be the best segment for the EZT test to be conducted to predict the BS test outcome. The *c*-statistics and Younden’s indices for the distal oesophagus also support this finding, showing the best diagnostic performance in the EZT for the distal oesophagus to distinguish between OCR and NCT in the BS test. The logistic regression of the EZT in the distal oesophagus with respect to BS tests for OCR (1) and NCT (0) is shown in Figure 5. The probability curve was computed to compare the odds of OCR or NCT occurring with respect to EZT. The minimal EZT of the distal oesophagus for odds of 1 (i.e., 50% OCR/50% NCT) was found at 3.4 min. This approximated finding supports the EZT threshold found during the ROC curve computation for the distal oesophagus at the maximum Youden’s index to predict between OCR and NCT in the BS study.

## 4. Discussion

This study validated the use of MII/pH sensors to measure oesophageal transit. This was through differentiating EZT between patients with significant dysphasia and no or minor dysphasia symptoms. Secondly, EZT successfully correlated to the BS test outcomes for NCT and OCR. Our study data were curated from 458 patients with treatment-naïve oesophagi, and the EZT tests were free from acid reflux interreference. The EZT data may not be normally distributed, as two-thirds of the patients had elevated HODQ scores in the validation for dysphagia, and approximately two-thirds of the BS test outcomes were NCT in the validation for oesophageal clearance. The EZT data being skewed for detecting dysphagia for poor clearance and predicting BS test outcomes and nullifying the normal distribution from the mean may thus lead to misinterpreting the mean averages and 95% confidence interval. The median and SQR of the EZT data were more accurate representations of the finding and, therefore, were reported in this study. The data comparisons were performed with using two-tailed *t*-tests on effective sample sizes for the type 1 error to be controlled at 5%. Overall, the statistical findings do support the validation of the EZT test be a clinically viable. We repeated our study using various statistical methods to confirm the findings.

The mode of assessing the dysphagia for the EZT test was by using the HODQ form, which quantitatively measures the dysphagia severity, and the questionnaire criteria fulfilled the participant profile as GI patients [12]. The first difference is the GI patients used in the creation of the HODQ form had lower-GI symptoms, and our participants had upper-GI symptoms (i.e., reflux symptoms and some patients having dysphagia symptoms). This essentially means our patient participants were being compared to asymptomatic patients of dysphagia and reflux symptoms. Therefore, the cut-off threshold of 6.4 for significant dysphagia outlined by the authors would be applicable. The authors did not derive the normal range HODQ scores from healthy volunteers, and their case group participants with dysphasia symptoms had HODQ scores of 3.4 at the 5th percentile. Thus, less than 10% of their case group population had HODQ scores of up to 6.4. This is a relatively small cohort of case-group dysphagia patients having normal HODQ scores, and our data of the EZT did not show a difference for HODQ scores between 0 and 6.4 or trends between the normal scores (see Figure 4b). We did observe the EZT increasing with higher HODQ scores from the 6.4 score threshold (see Figure 4c). We also found the EZT of the distal oesophagus in order to successfully differentiate between normal and elevated HODQ scores (EZT test sensitivity and positive predictive value probabilities of elevated HODQ scores for poor transit were 81% and 84%, respectively).

The HODQ form assesses patients’ oropharyngeal dysphagia symptoms by evaluating choking and coughing on swallowing and the sensation of food stuck in the throat. The chest symptoms or retrosternal dysphagia could be also be assessed. However, the HODQ form does not distinguish separate cut-off threshold scores for oropharyngeal dysphagia and retrosternal dysphagia or the scores to distinguish the types of dysphagia. This could have been compared to the specific impedance sensors in the proximal and distal oesophagus that were measuring the transit to explain the level of dysphagia (oropharyngeal or retrosternal). The HODQ form also assesses the dysphagia from bolus types like solids, semi-solids and liquids. There are no thresholds in the HODQ scores to correlate dysphagia to the bolus type (i.e., dysphagia to solids only, dysphasia to both solids and liquids, etc.). This could be useful as EZT has not been assessed or validated with dysphagia for semi-solids and solids. The HODQ form is simple and basic, whilst dysphasia is a complex condition and has multi factorial aetiologies. The oesophageal (or retrosternal) dysphagia symptoms can be a manifestation of anatomical abnormalities (i.e., oesophageal diverticulum, corkscrew oesophagus, etc.) or dysfunctional oesophagus from oesophageal body motility disorders or impaired GOJ relaxation. The creators and validators of HODQ scoring for dysphagia severity did not study the HODQ scores for the aforementioned oesophageal pathologies [12]. Although the authors report some of their participants had HRM studies, there is no report on the oesophageal motility disorders and the HODQ scores for the motility disorders. This would limit the EZT predictability in these oesophageal pathologies from HODQ scores.

A total of 151 of the 311 patients (48.6%) with NCT on the BS test had elevated HODQ scores for significant dysphagia and would be classified as having functional dysphagia. However, oesophageal transit on MII/pH sensors was able to identify the causes of dysphasia in 88 patients (58.3%), which were from poor oesophageal clearance as opposed to oesophageal retention (distal oesophageal clearance exceeding 1 min but not reaching the 3.7 min threshold). One possible reason why the EZT test was able to explain the dysphagia may be from the technical difference in measuring the oesophageal transit between MII/pH sensors and the BS test. The EZT technique quantitatively measures the oesophageal transit time between the flow rate of oesophageal filling and clearance, whereas the BS test is a qualitative measurement of either a positive or negative finding for oesophageal retention of the barium sulphite contrast. The dysphasia in these patients was not caused by oesophageal retention (or stenosis) but rather from poor oesophageal emptying. The complete oesophageal retention (OCR in the BS test) in our study correlates the EZT threshold at 3.7 min (test sensitivity and PPV of OCR were, respectively, 79% and 83%). We assessed this and found it in numerous statistical methods. To better understand the conditioning for OCR in the BS test, the oesophagus is filled with 200 mL of barium sulphite contrast, which has a substance density of 4.3 g/cm^3^. In a fully loaded oesophagus with barium contrast, the fluid weight of 8.83N along with peristaltic propagation waveforms are being resisted from traversing across the restricted GOJ. The OCR in the BS test is a severe form of poor oesophageal transit, where there is no oesophageal clearance, and there is a higher degree of EZT found. We wondered if the technical analysis of the BS study could only offer EZT validation for oesophageal retention or OCR and not of poor oesophageal clearance associated with dysphagia. Then, we wondered whether the newer BS technique, called timed the BS study, would be more suitable and appropriate to validate the use of MII/pH sensors for oesophageal transit tests. Unlike the standard BS test performed on our patients, the timed BS test (like the EZT test) quantitatively and simultaneously measures the oesophageal columnar height and clearance time [9]. It would be of clinical interest to see how close the timed BS test is to the 1 min threshold in patients with elevated HODQ scores. Unfortunately, the timed BS test is not yet widely performed but seems to be promising as oesophageal emptying is being measured, which would explain the dysphagia with poor oesophageal clearance. This would be a better validator of the MII/pH sensors measuring oesophageal transit in dysphagia patients.

Researchers have found oesophageal transit varies from the viscosity of the substance [2], and the viscosity of barium sulphite could vary between BS tests. In clinical practice, the contrast is found in dry powder form, and water is added to make the paste. One may argue that the viscosity of the barium contrast plays no role if there is simply complete stenosis of contrast in the oesophagus (i.e., in the standard BS test). In addition, the influence of contrast viscosity may play a role if the dysphagia symptom is caused by poor oesophageal clearance, or slow emptying of the oesophagus in the case of the timed BS test. Saline (0.9% *w/v* NaCl) has a viscosity of 1, but the barium contrast viscosity varies by the preparation and also contains other ingredients to enhance its property as a radiographic agent. The BS test transit and the viscosity of the contrast require further research to determine normal ranges for standardising barium contrast viscosity for clinical practice. The use of barium contrast on impedance sensors to measure oesophageal transit has been investigated but posed a difficulty because the barium contrast residue was left smeared on the impedance sensors [7,8], and it was difficult to ascertain the oesophageal clearance or retention. In contrast, the ionicity of saline (0.9% *w/v* NaCl) was significantly lower than the oesophageal mucosa, and the saline transit was easily identified from the difference in alternating current [3,11]. Researchers have used mixed barium contrast with saline as a single transit marker in an attempt to capture the alternating current of saline from mucosa [7,14]. However, the salt concentration is diluted by the mixing, and a decrease in the alternating current may simply result in the new mix substance having similar impedance to the mucosa. As a consequence, the measuring of saline transit cannot be easily interpreted within the oesophageal lumen. This study did not measure the impedance (or alternating current ranges) of saline intra-oesophagus as part of the validation process measuring EZT. This was not necessary, as the alternating current of uncontaminated saline was significantly lower than the oesophageal mucosa in our patient participants who did not have oesophagitis (see Figure 3). However, the mucosal impedance (or alternating current) in patients with oesophagitis has been reported as low as 465 Ω [5], and the baseline mucosal impedance as low as 886 Ω in patients with major motility disorders from residual bolus intra-oesophagus (e.g., achalasia) [4]. The low oesophageal baseline impedance in patients in these oesophageal pathologies may pose some difficulty reading the EZT, as there would be little change in the alternating current between mucosa and the saline. The validation of MII/pH sensors measuring EZT at low oesophageal impedance (or closely to the saline impedance) has not been investigated in this paper. But the key principles apply in measuring the EZT at low oesophageal impedance, which is to assess for the changes in the alternating current on impedance sensors that would manifest in antegrade pattern between the impedance sensors. As the baseline impedance is low in this cohort of patients, the duration between the 50% impedance drop to 50% recovery may be underestimated, which may require separate validation in patients with oesophagitis or with major motility disorders.

The HRMZ study was not used for validating the EZT during MII/pH tests. This was because the HRMZ test is a stationary test for measuring oesophageal motility [15] and is not ideal for measuring prolonged retention found in major motility disorders. The MII/pH test involves 24 h monitoring, which can measure prolonged retention, and the current study found 35 patients (7.64%) with EZT exceeding one hour in oesophageal retention. The impedance on HRMZ screening is viewed as a purple contrast colour that is subjective to interpretation of the oesophageal transit. In contrast, the EZT recording in the MII/pH test is a simple graphical representation of saline transit that is easily identifiable by the significant difference in the alternating current between mucosa and saline (see Figure 3). This study showed the time lapse in the alternating current for saline to be a measure of oesophageal transit. The HRMZ screening in the impedance trace mode can show graphical presentation of the impedance changes during the saline transit, but the manometric sensors integrated on the HRMZ catheter that produce pressure waveforms have been reported to obscure the impedance trace that measure the oesophageal transit [8]. The MII/pH catheter does not have manometry sensors and is excluded from this pitfall. The MII/pH catheter also has the advantage with its pH sensors that it can identify reflux events interfering with the oesophageal transit study. The current study identified 11 EZT tests (0.911%) with acid reflux interference. The HRMZ catheter does not have integrated pH sensors to detect acid reflux during the oesophageal transit testing, and on occurrence this would be perceived as longer oesophageal transit or as retention. This study did not include the HRM test finding to validate use of MII/pH sensors for oesophageal transit. The HRM device is used for assessing oesophageal motility and not transit. The dysphagia symptom is not necessarily caused by dysmotility (which HRM measures) but from poor oesophageal clearance or retention of bolus (which EZT and the BS test measure).

The MII/pH study does present a limitation by not being able to detect regurgitation of saline or non-acid reflux during the EZT test, which could take up to another minute to clear from the oesophagus [11] (if it occurs during the EZT test) and may be perceived as prolonged EZT. Another limitation of the EZT measurements is in patients with aerophagia, which presented in two phenomena during the EZT test. First, the complete transmission of air from the oesophagus to the stomach with saline was identified by a progressive single impedance waveform (>10,000 Ω). The EZT could still be interpreted in this scenario, as impedance sensors detected changes of alternating current between mucosa and saline. The second phenomena was from stagnation of air in the oesophageal segments, and impedance sensors could not detect changes in the alternating current between mucosa and saline, and the segmental EZTs were not always interpretable (see Table 7). The risk of aerophagia was minimised from our protocol by requesting patients to inhale and rapidly drink saline through a straw. We did still observe air stagnating in the proximal and mid oesophageal segments in 32 patients (6.99%) by detecting prolonged high impedance. Aerophagia/air stagnation interrupting the oesophageal transit testing in HRMZ studies was found in up to 38% of patients [4]. In our cohort of patients, the impedance signal of saline was only masked by the air accumulating in the proximal and mid oesophagus and not in the distal oesophagus. The saline retention in the distal oesophagus was still easily identifiable by the alternating current of saline (<500 Ω) and mucosa (>2000 Ω). This study suggests that air was stagnating above the saline fluid, and this could be supported by the BS test with OCR findings.

## 5. Conclusions

The findings of this study showed that MII/pH sensors were able to measure oesophageal transit without posing additional risk or burden to patients. The EZT test was simple to perform and can easily be incorporated into the MII/pH investigation. There is a need to measure oesophageal transit in parallel to oesophageal motility in order to fulfil the latest Chicago Classification guideline, which MII/pH sensors can cater to. The impedance and pH sensors of the distal oesophagus seem to be in the optimal location to conduct the EZT test, which also excludes interreference from acid reflux. The EZT of the distal oesophagus exceeding the threshold of 1 min is affiliated with dysphagia symptoms from poor oesophageal clearance, and thresholds exceeding 3.7 min are predictive of OCR in the BS test.

## Figures and Tables

**Table 1:** Study inclusion and exclusion criteria.

*Study Inclusion Criteria*	*Study Exclusion Criteria*
Patients referred for standard oesophageal diagnostic testing (including HRM and MII/pH studies). Upper GI endoscopy, BS test and HRM to be undertaken before the MII/pH test. MII/pH test is performed within 12 months of the upper endoscopy and the BS test. Adult patients invited for this study (male and female, 18 to 100 years of age). Patients having the mental capacity to consent for the EZT test during their MII/pH test. Patients off prokinetic therapy for at least 1 week (including antibiotics). No history of upper GI surgery, thoracic surgery or upper abdominal surgery. Patients filling out the Hospital Odynophagia Dysphagia Questionnaire (HODQ) form within 6 months of the MII/pH test.	Uncontrollable hypertension, chronic renal disease or unstable cardiac condition. Previous surgical history in the thorax, oesophagus and the stomach. Reflux oesophagitis and/or Barrett’s oesophagus found on upper endoscopy. Eosinophilic oesophagitis observed on histology screening. Oesophageal mass found or suspected on upper endoscopy or on the BS test. Oesophageal diverticulum was found or suspected on upper endoscopy, BS test or on the HRM test. Patients poorly tolerating the MII/pH catheterisation or failing the intubation of the HRM catheter or the MII/pH catheter. Patient swallows of saline volume (200 mL) exceeding 20 s. Oesophageal pH sensor detecting acidic pH levels (≤4) during the EZT study. Patient on prokinetic therapy or antibiotics during the MII/pH test.

**Table 2:** EZT of the proximal, mid and distal oesophageal segments in patients with normal and elevated HODQ scores, and in the BS test outcomes (data are presented in minutes for the median [25th–75th quartile] range).

Oesophageal Segment	Normal HODQ Scores (N = 163)	Elevated HODQ Scores (N = 256)	NCT (N = 311)	OCR (N = 147)	* *p*-Value	** *p*-Value
**Proximal**	0.23 [0.15–0.40]	0.47 [0.24–2.00]	0.26 [0.17–0.45]	5.75 [0.49–20.8]	<0.001	<0.001
**Mid**	0.31 [0.17–0.62]	0.85 [0.34–6.10]	0.36 [0.36–0.72]	15.4 [1.53–41.6]	<0.001	<0.001
**Distal**	0.57 [0.28–1.00]	1.90 [0.85–17.0]	0.72 [0.35–1.40]	22.8 [5.30–55.0]	<0.001	<0.001

* p-value comparing EZT in patients with normal and elevated HODQ scores. ** *p*-value comparing EZT in patients with NCT and OCR on BS tests.

**Table 3:** Showing EZT with respect to the BS tests in patients asymptomatic of dysphagia (HODQ scores 0), with minor dysphagia (HODQ scores > 0 and < 6.4) and significant dysphagia (HODQ scores > 6.4).

	Barium Swallow Test Outcome			
	NCT	OCR	*t*-Statistic	*p*-Value
**Asymptomatic Patients (N = 67)**				
Proximal oesophagus	0.18 [0.12–0.37]	0.29 [0.25–0.31]	0.214	0.831
Mid oesophagus	0.26 [0.14–0.50]	0.39 [0.33–0.41]	0.369	0.714
Distal oesophagus	0.40 [0.20–0.82]	0.57 [0.44–0.57]	0.349	0.728
**Minor Dysphagia (N = 96)**				
Proximal oesophagus	0.23 [0.16–0.38]	2.00 [0.37–7.58]	3.62	<0.001
Mid oesophagus	0.30 [0.17–0.62]	3.85 [1.11–10.1]	2.16	0.033
Distal oesophagus	0.52 [0.28–1.00]	13.1 [3.28–31.5]	3.19	0.002
**Significant Dysphagia (N = 256)**				
Proximal oesophagus	0.28 [0.20–0.49]	6.70 [0.68–21.5]	6.63	<0.001
Mid oesophagus	0.48 [0.27–0.88]	18.8 [2.50–47.5]	7.00	<0.001
Distal oesophagus	1.00 [0.51–1.90]	26.3 [6.33–60.0]	8.29	<0.001

**Table 4:** Showing the EZT cut-off thresholds for saline clearance in the proximal, mid and distal oesophagus and significant dysphagia symptoms.

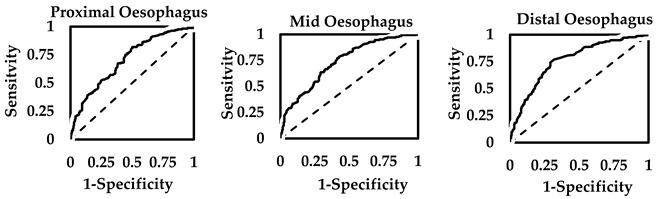								

Oesophageal Segments	EZT Cut-Off Threshold (mins)	Sensitivity	Specificity	PPV	NPV	LR+	OR	*c*-Statistics
**Proximal**	0.47	0.50	0.83	0.81	0.53	2.92	4.82	0.71
**Mid**	0.66	0.57	0.79	0.80	0.55	2.69	4.94	0.73
**Distal**	1.00	0.81	0.75	0.84	0.72	3.32	13.6	0.76

**Table 5:** Comparison of EZT tests between patients asymptomatic of dysphagia (HODQ scores 0) and patients with minor dysphagia (HODQ scores from >0 to 6.4).

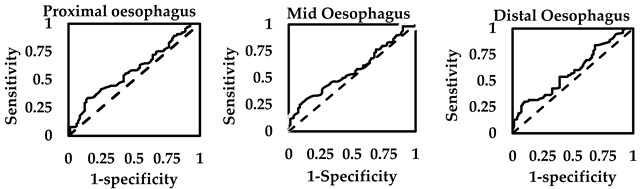								

Oesophageal Segments	EZT Cut-Off Threshold (mins)	Sensitivity	Specificity	PPV	NPV	LR+	OR	*c*-Statistics
**Proximal**	0.13	0.85	0.34	0.62	0.65	1.29	2.94	0.581
**Mid**	0.15	0.86	0.30	0.63	0.61	1.22	2.59	0.578
**Distal**	0.22	0.90	0.30	0.63	0.70	1.29	3.99	0.591

**Table 6:** EZT cut-off thresholds to predict OCR and NCT on the BS test for the proximal, mid and distal oesophagus.

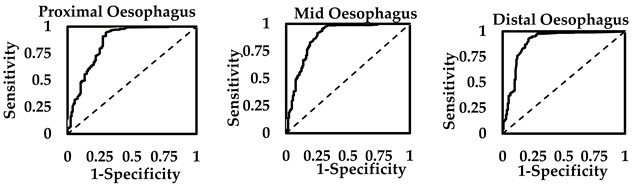								

Oesophageal Segments	EZT Cut-Off Threshold	Sensitivity	Specificity	PPV	NPV	LR+	OR	*c*-Statistics
**Proximal**	0.59	0.73	0.85	0.68	0.88	4.89	15.5	0.85
**Mid**	1.9	0.74	0.92	0.80	0.89	9.18	33.0	0.88
**Distal**	3.7	0.79	0.93	0.83	0.91	10.6	46.2	0.89

**Table 7:** Detailing the segmental EZT test cases that were not interpretable in the proximal and mid oesophageal regions owing to residual air stagnating intra-oesophagus (n = 32).

	NCT	OCR
Residual air stagnating in the proximal oesophagus only	4	9
Residual air stagnating in the proximal and mid oesophagus	9	10

## Data Availability

This project is based on patients’ clinical data, and we obtained approval to publish data of the analysed results to draw conclusions, which can be found in this manuscript. The raw clinical data are not available to the general public.
